# Efficacy of Omentopexy on Complications of Laparoscopic Sleeve Gastrectomy

**DOI:** 10.1007/s11695-024-07363-6

**Published:** 2024-06-24

**Authors:** Yalcin Burak Kara, Yahya Ozel, Samet Yardimci

**Affiliations:** 1 General Surgery Department, Bahcesehir University VM Medical Park Pendik Hospital, Fevzi Çakmak Mahallesi, D100, Cemal Gürsel Cd. No:9, Pendik, 34899 Istanbul Turkey; 2 General Surgery Department, Dogus University VM Medical Park Pendik Hospital, Fevzi Çakmak Mahallesi, D100, Cemal Gürsel Cd. No:9, Pendik, 34899 Istanbul Turkey; 3 General Surgery Department, Istinye University VM Medical Park Pendik Hospital, Fevzi Çakmak Mahallesi, D100, Cemal Gürsel Cd. No:9, Pendik, 34899 Istanbul Turkey

**Keywords:** Laparoscopic sleeve gastrectomy, Bleeding and leakage, Omentopexy, Staple-line imbrication

## Abstract

**Background:**

Laparoscopic sleeve gastrectomy (LSG) is a commonly performed type of bariatric surgery. Early complications of LSG include bleeding, leakage, pulmonary embolism, and surgical site infections. Most surgeons try to implement preventive methods, such as omentopexy. Staple line-imbrication, which has a difficult learning curve, often prevents complications. This study aimed to evaluate the effect of omentopexy on patients with imbricated LSG.

**Material and Methods:**

The study applied a retrospective data analysis design to patients who underwent LSG between 2020 and 2023. All patients’ staple lines were imbricated, and patients were then divided into two groups: omentopexy group and control group. Patients’ demographic features, such as age, gender, height, weight, body mass index(BMI), bleeding, leakage, and reoperations, were recorded and examined retrospectively.

**Results:**

A total of 1356 patients were included in the study (540 in omentopexy, 816 in control), of which the mean age was 37.9 ± 10.5 years, 82.3% were women, and mean BMI was 40.9 ± 5.8 kg/m^2^. The mean bleeding rate was 1.0% (1.3–0.7%), the mean leakage rate was 0.2% (0.2–0.2%, respectively), and the mean reoperation rate was 0.6% (0.7% and 0.5%, respectively). No statistically significant differences were observed.

**Conclusion:**

Omentopexy is a technique that is widely used to prevent staple line complications. According to our study, omentopexy applied to an imbricated stapler line increased the operation time but did not affect bleeding or leakage ratios. This is the first study to evaluate the effect of omentopexy on imbricated staple lines. The findings of the study indicate that omentopexy has no additional benefit on early complications when using staple-line imbrication.

**Graphical Abstract:**

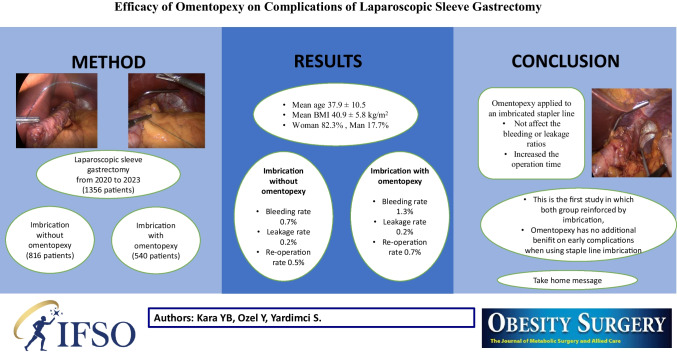

## Introduction

Obesity is a chronic disease that affects many bodily systems and is associated with multiple comorbid disorders. It is accepted as a global epidemic issue. One of the best long-term solutions to this health problem is bariatric surgery. Laparoscopic sleeve gastrectomy (LSG) is the most commonly performed type of bariatric surgery, accounting for nearly 61% of all bariatric surgeries globally [1, 2, 3, 4].

Early complications of LSG include bleeding, staple line leak, intra-abdominal abscess, wound infection, pulmonary thromboembolism, or partial spleen infarction [5] Bleeding is one of the most common reasons for post-LSG reoperation. Various studies have found that the rate of post-LSG bleeding risk ranges from 0.38 to 9% and that the rate of required reoperation is between 0.16 and 1%. The overall mortality rate was attributable to bleeding in approximately 1% of all bleeding cases. Bleeding can occur intra-abdominally or intraluminally, and the source of bleeding is typically the staple line, trocar site, dissected omental tissue, or damaged spleen or liver. Post-LSG bleeding is also associated with complications such as pneumonia, cardiac disease, surgical site infection, acute kidney injury, pulmonary embolism, reoperation, and mortality [6, 7, 8, 9]. This knowledge explains the importance of the subject and why it tends to attract the attention of surgeons.

An additional complication and the most alarming is postoperative leakage. Various studies have found leakage rates ranging from 1.1 to 3.6%. This complication can lead to long hospital stays, repeat endoscopic interventions, repeat laparoscopy and bypass conversions, extended antibiotic usage, intensive care stays, and mortal complications [10, 11].

Long-term complications of LSG include gastroesophageal reflux disease (GERD), mid-sleeve stricture, twisting, and nutritional deficiencies [3].

Most surgeons choose a particular reinforcement method (e.g., omentopexy) to prevent these complications. A meta-analysis showed that compared to LSG with no reinforcement, sleeve gastrectomy with omentopexy decreased the complication rates of GERD, twisting, and gastrointestinal symptoms. Although the leakage rate associated with LSG surgeries reinforced with omentopexy is lower than that of LSG with no reinforcement, there is no significant difference in postoperative bleeding [3, 12].

Omentopexy, which involves attaching the greater omentum to the sleeve stapler line, can be achieved using different techniques in different centers or by different surgeons. There is currently no consensus on the best suture type (prolene suture or V-LOC), technique (continue or interrupted), fixed part (proximal, distal, or whole sleeve line), or which part of the omentum should be used (free edge or gastrocolic ligament).

Bleeding from the stapler line can be managed using different approaches. Unreinforced stapler lines are the most vulnerable to postoperative bleeding, but reinforcement can be achieved by suture oversewing or imbrication, the use of buttress material or an absorbable polymer membrane, or by performing an omentopexy. Most surgeons (79%), as recommended by various guidelines, prefer to use one of these reinforcement methods to manage and decrease the risk of postoperative bleeding [8, 10, 13, 14, 15]. In our clinic, suture imbrication was used to reinforce the staple line during LSG.

Most studies and meta-analyses have compared laparoscopic sleeve gastrectomy with either omentopexy or no reinforcement [3, 12]. To our knowledge, our study is the first to assess the use of imbrication with all sleeve gastrectomy patients in both groups. The aim of this study was to evaluate the effect of omentopexy with suture imbrication on post-LSG complications.

## Methods

### Patients

This study was designed as an observational cohort study, and all data were analyzed retrospectively. All patients who underwent LSG between 2020 and 2023 were examined. The patients’ demographic features, such as gender, age, height, weight, and body mass index, were documented. The patients’ biochemical laboratory tests and radiologic evaluations were analyzed. Hospital records were used to examine patient reoperation, rehospitalization, and endoscopic evaluation data.

### Inclusion Criteria

Patients who underwent LSG had a body mass index (BMI) between 30 and 65 kg/m^2^ and were between 14 and 70 years old.

### Exclusion Criteria

Patients who underwent gastric re-sleeving or gastric sleeve revision (gastric band-to-sleeve gastrectomy), who had a previous upper abdominal open surgery, who were not able to perform an imbrication suture, or who had previous usage of blood thinning medication.

**Fig. 1:**
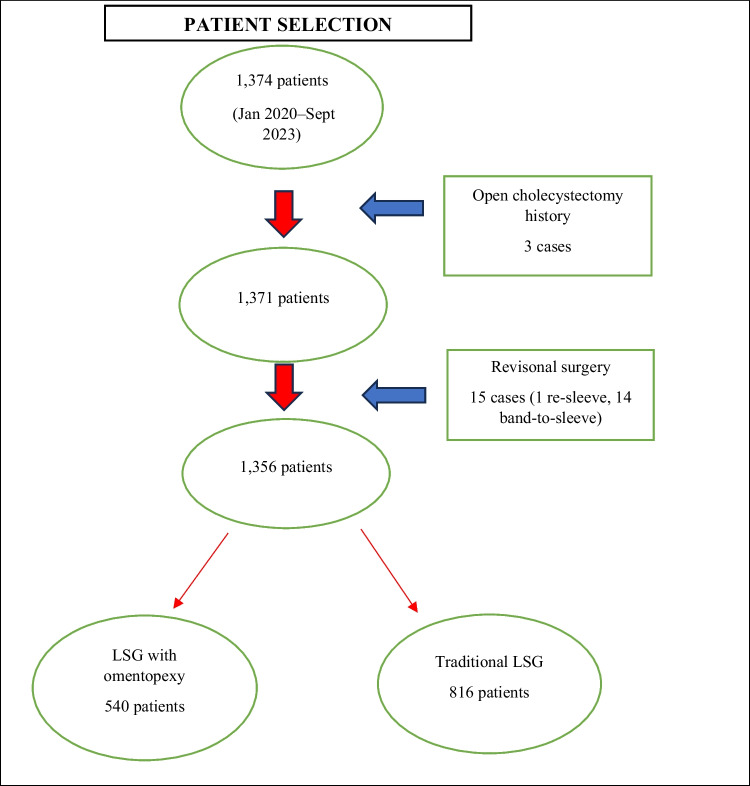
Patient selection and exclusion process

A total of 1374 patients who underwent LSG were examined. Three patients were excluded due to a history of upper abdominal open surgery (open cholecystectomy), and 15 patients were excluded due to revisional sleeve surgery (one re-sleeving gastrectomy and 14 gastric band removals or revisional sleeve gastrectomies). After exclusion, 1356 patients were approved for inclusion and, according to whether they underwent omentopexy or not, they were assigned to one of two groups: the control group (traditional LSG) and the omentopexy group (Fig. 1).

For retrospective studies such as this type of study, formal consent or ethical approval is not required. Informed consent does not apply.

### Surgical Technique

#### Control Group: Traditional LSG

All patients underwent five-trocar laparoscopies. After insufflation of the abdomen, the greater curvature of the stomach was dissected from the nearby pylorus to the left diaphragmatic area. After the fundus was fully mobilized, a 39-Fr calibration tube was placed in the stomach, and the stomach was then separated by stapling, beginning 2–4 cm away from the pylorus, and continuing until 1 cm away from the esophagogastric junction.

Covidien® Tri-Staple™ and Endo-GIA™ with a manual handle were preferred for surgical resection. The type of cartridge choice was decided by to thickness of the stomach. The resection was completed by using Endo GIA™ black reload with Tri-Staple™ in the antrum, Endo GIA™ purple reload with Tri-Staple™ in the body, and Endo-GIA™ blue cartridge in the remaining part, respectively. After the resection was completed, all sleeve staple lines were imbricated continuously using 2/0 V-Loc sutures. An intra-operative leakage test was performed with methylene blue. After controlling the trocar site, which was not routinely closed, the operation was completed. No routine drains or nasogastric tubes were used [16].

#### Omentopexy Group

All patients in this group were operated on according to the same procedure as those in the control group. After the leakage test was performed, the omentum, which was separated from the greater curvature of the stomach, was fixed with at least 4 or 5 2/0 V-loc sutures, starting with the proximal part of the imbricated sleeve line.

### Postoperative Management

To prevent thromboembolic complications, Enoxaparin (4000 IU) was administered nightly to every patient from operation day to post-operative 20 days.

### Bleeding and Leakage Criteria

If a patient presented with tachycardia, abnormal abdominal pain, abnormal sweating, dizziness, or bloody vomiting, hemoglobin and hematocrit (Hct) levels were examined. In cases of decreased Hct levels, the abdomen was further assessed with a CT scan. If patients had decreased Hct levels and hematomas were visible on the CT scan, these cases were classified as “bleeding cases.” Patients whose CT scans showed fluid-air collection near the sleeve line, which were then verified endoscopically, were classified as “leakage cases.”

### Study Objectives

The primary objective of this study was to evaluate whether imbrication with omentopexy would impact post-LSG bleeding risks in imbrication patients. The secondary objectives were to compare the bleeding and leakage rates in our clinic data and in the literature.

### Statical Analysis

The mean standard deviation, median, minimum–maximum, frequency, and ratio values were used to obtain descriptive statistics. Variable distribution was measured using the Kolmogorov–Smirnov test. The Mann–Whitney U test and independent sample t-test were used to analyze quantitative independent data, the chi-square test was used to analyze qualitative independent data, and the Fischer test was used when chi-square test conditions were not met. The SPSS 28.0 program was used to conduct these analyses.

## Results

The study included 1356 patients with a mean age of 37.9 ± 10.5 years and a mean BMI value of 40.9 ± 5.8 kg/m^2^, of whom 82.3% were women. Among these patients, the bleeding rate was 1.0%, the leakage rate was 0.2%, the trocar site hernia rate was 0.2%, and the reoperation rate was 0.6%. The patients’ demographic data, including age, gender, weight, height, BMI, leakage rate, bleeding rate, trocar site hernia ratio, and reoperation rate values, are shown in Table 1.

**Table 1:** Patient demographics

		Min–Max	Median	Mean ± SD/*n*-%		
Age		15.0–70.0	37.0	37.9	±	10.5
Gender	*Woman*			1116		82.3%
	*Man*			240		17.7%
Weight		54.0–204.0	109.8	113.0	±	20.6
Height		144.0–195.0	165.0	165.9	±	8.2
BMI		21.6–64.0	40.3	40.9	±	5.8
Bleeding	( −)			1,343		99.0%
	( +)			13		1.0%
Leakage	( −)			1,353		99.8%
	( +)			3		0.2%
Reoperation	( −)			1,348		99.4%
	( +)			8		0.6%

*Min–Max*, minimum–maximum; *SD*, standard deviation; *BMI*, body mass index

**Fig. 2:**
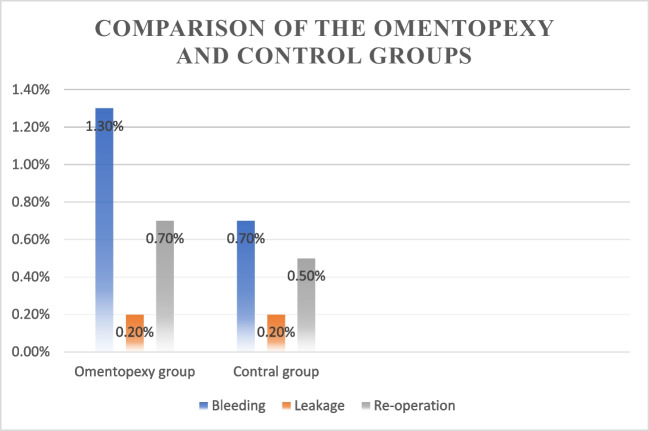
Comparison of groups

The patients were stratified according to whether or not they underwent omentopexy and then assigned to two groups: the omentopexy group (540 patients) and the control group (816 patients). The mean age of the patients in the omentopexy group was 38.6 ± 10.6 years, which was significantly higher than that of the control group. The mean BMI value of the omentopexy group was 41.6 ± 6.2, which was also significantly higher than that of the control group (*p* < 0.05). The bleeding ratio of the omentopexy group (1.3%) was higher than that of the control group (0.7%); however, there was no significant difference in the bleeding ratio between the two groups (*p* > 0.05). Both groups had a leakage rate of 0.2%; thus, there was no significant difference between the two groups for these values (Table 2, Fig. 2). Omentopexy and control group demographic data and a comparison of the two groups are shown in Table 2.

**Table 2:** Comparison of the omentopexy and control groups

		Control group				Omentopexy Group				*p*	
		Mean ± SD/*n*(%)			Median	Mean ± SD/n(%)			Median		
Age		37.5	±	10.4	36.5	38.6	±	10.6	38.0	**0.046**	^m^
Gender	*Woman*	653		80.0%		463		85.7%		**0.007**	^X2^
	*Man*	163		20.0%		77		14.3%			
Weight		111.9	±	20.4	108.0	114.8	±	20.8	111.0	**0.004**	^m^
Height		165.8	±	8.1	165.0	166.0	±	8.4	165.0	0.714	^m^
BMI		40.5	±	5.5	40.1	41.6	±	6.2	40.9	**0.001**	^m^
Bleeding	( −)	810		99.3%		533		98.7%		0.299	^X2^
	( +)	6		0.7%		7		1.3%			
Leakage	( −)	814		99.8%		539		99.8%		1.000	^X2^
	( +)	2		0.2%		1		0.2%			
Reoperation	( −)	812		99.5%		536		99.3%		0.555	^X2^
	( +)	4		0.5%		4		0.7%			

^X2^chi-squared test; ^m^ Mann–Whitney *U* test

The concomitant surgeries, categorized as hiatal hernia repair (4.2%), laparoscopic cholecystectomy (3.6%), and hiatal hernia repair with laparoscopic cholecystectomy (0.2%), are shown in Table 3. There were no bleeding or reoperation cases among the concomitant surgeries. In one case of leakage, the patient had undergone simultaneous hiatal hernia repair.

**Table 3:** Concomitant surgery

Concomitant surgery	*n*	%
( −)	1247	91.9%
( +)	109	8.1%
Laparoscopic cholecystectomy	49	3.6%
Hiatal hernia repair	57	4.2%
Hiatal hernia repair + laparoscopic cholecystectomy	3	0.2%

## Discussion

Obesity is a worldwide problem, and the most effective solution is bariatric surgery. LSG is the most frequently performed bariatric surgical procedure, at nearly 61% of all bariatric surgery in the USA [1, 2, 4].

Almost all the steps of sleeve gastrectomy are standardized, and LSG is performed by each surgeon in the same manner, but there is currently no consensus on the best methods for reinforcing the sleeve line to decrease bleeding and leakage rates. Some surgeons do not use any reinforcement method, but most surgeons opt for one of several methods that include imbrication with suture, oversewing with suture, using bovine pericardium, and using an absorbable membrane. Due to the ease of the procedure, omentopexy is one of the most commonly used reinforcement methods [1, 2, 10].

In addition to preventing postoperative complications, omentopexy is also used to improve gastrointestinal symptoms, such as nausea and vomiting and gastroesophageal reflux symptoms, during early postoperative recovery. Some surgeons have also suggested that omentopexy decreases the rate of staple line twisting and thoracic herniation of the stomach over the long term [17, 18, 19].

The aim of this study was to reveal the effect of omentopexy on the imbricated staple line of LSG patients and to evaluate its relationship with early postoperative complications.

Different surgeons describe omentopexy using different techniques. Some surgeons prefer continuous stitches, while others prefer separate stitches. Some surgeons complete the omentopexy with three separate stitches, and some with five stitches. The common goal of all these surgeons is to reduce bleeding and leakage risks while affixing the stomach to the greater omentum [18, 19, 20, 21].

A meta-analysis of 17 studies suggested that reinforcing the staple line with oversewing decreased postoperative bleeding significantly when compared to the absence of reinforcement (0.86% versus 4.83%) [22]. In a study of 98,142 patients, Zafar found a postoperative bleeding rate of 0.80% with no reinforcement, 0.60% with suture oversewing, and 0.56% with buttressing material. Reinforcement of the staple line significantly reduces postoperative bleeding [8]. Our study identified a postoperative bleeding rate of 1%, which aligns with the rates indicated in the literature (Table 1).

Most published studies have compared omentopexy with reinforced staple lines to omentopexy with unreinforced staple lines. One study of 2000 patients, 1000 with omentopexy and 1000 with no reinforcement, suggested the positive effects of omentopexy on early postoperative complications. Another study of 3942 patients, divided into three groups (no reinforcement, reinforcement with fibrin glue, and reinforcement with omentopexy), found that omentopexy decreased postoperative bleeding and leakage [17]. Our study compared two reinforced staple lines and is, to the best of our knowledge, the first study to compare LSG with imbrication and LSG with imbrication plus omentopexy. Aside from the omentopexy group showing a higher postoperative bleeding rate (1.3%) than the control group (0.7%), there were no significant statistical differences between the two groups.

Different studies have suggested that the post-LSG leakage rate ranges from 1.1 to 5.4% and that the resulting overall mortality rate is 0.4%. The high rate of this serious complication makes LSG leakage the most dangerous [10, 23, 24]. The post-LSG leakage rate in our study was 0.2% for both the omentopexy and control groups (Table 1). The lower leakage rate may be explained by sleeve staple-line imbrication, which is a safe and reliable method. Our findings suggest that when staple-line imbrication is used for reinforcement, omentopexy is not required to prevent leakage.

Regarding post-LSG reoperation, a single-center study of 664 patients found that only 0.5% required reoperation, while 2% of the patients experienced postoperative bleeding (3 patients in 13 bleeding cases). Only three patients required reoperation and the most preferred conservative management [25]. Another study of 612 LSG patients found a post-LSG reoperation rate of 0.4%. In this study, seven patients required an additional laparoscopy due to leakage (5 cases), twisting (1 case), or bleeding at the staple line (1 case). All laparoscopic operations were completed safely [26]. Another study of 1860 LSG patients showed that 20 (1.1%) experienced post-LSG hemorrhaging and 11 required reoperation (0.6%) [9]. In our study, the postoperative bleeding rate was 1%, and the reoperation rate was 0.6% (Table 1). The high reoperation rate may be due to conservatism and avoiding the risk of abscess formation due to hematomas accumulating near the stomach staple line. Regarding hemorrhage, there is no consensus in the literature, and our approach was to reoperate if CT scans of a hemodynamically stable patient revealed a hematoma near the stomach staple line.

A consensus on surgeries performed at the same time as LSG is also absent from the literature. Despite some surgeons making decisions based on the presence of a hiatal hernia and preferring bypass surgeries when a hiatal hernia is observed via endoscopy, the tendency of some bariatric surgeons is to perform LSG with hiatal hernia repair during the same operation. Few studies have evaluated whether laparoscopic cholecystectomy is safe when performed at the same time, and although hiatal hernia repair is safe, there is controversy about its long-term effects on gastroesophageal reflux [27, 28, 29, 30, 31]. In our study, laparoscopic cholecystectomy was performed simultaneously with LSG in 3.6% of patients, hiatal hernia repair was performed simultaneously with LSG in 4.2% of patients, and both methods were performed simultaneously with LSG in 0.2% of patients (Table 3). Despite increasing the surgical dissection area, no excessive bleeding was encountered. In one leakage case, the patient underwent hiatal hernia repair and omentopexy. No abnormal steps that might cause leakage due to hiatal hernia repair were observed during the repeated review of the surgical video. Based on our professional experience, we recommended that patients with BMIs below 50 kg/m^2^ undergo simultaneous surgery when a preoperative endoscopic examination revealed a hiatal hernia or asymptomatic gallstones.

## Study Limitations

Despite the study’s large sample size and the use of a control group, it was designed as a single-center retrospective analysis. If it had been designed as a multi-center and prospective study, the results might have yielded more significant results pertaining to the effect of omentopexy on post-LSG complications. In our study, the bleeding rate of the omentopexy group was higher than the control group, but there were no statistically significant results (Fig. 2). Multicenter design and a bigger sample size may lead to significant results.

Furthermore, the complication rates in our study were low, so despite the lower bleeding rate in the control group, there was no statistical difference between the two groups. We think that low-risk rate in the control group in our study is due to the effect of imbrication stitches.

## Conclusion

Omentopexy has been shown to decrease post-LSG leakage and bleeding rates and improve patients’ gastrointestinal symptoms; however, our study found that omentopexy did not affect bleeding or leakage rates in patients who underwent staple-line imbrication. In addition, performing an omentopexy prolonged the operation time. Omentopexy has no additional benefit on early complications when using staple-line imbrication.

## References

[1] English WJ, DeMaria EJ, Brethauer SA, Mattar SG, Rosenthal RJ, Morton JM. American Society for Metabolic and Bariatric Surgery estimation of metabolic and bariatric procedures performed in the United States in 2016. Surg Obes Relat Dis. 2018;14:259–63. 29370995 10.1016/j.soard.2017.12.013

[2] Eisenberg D, Shikora SA, Aarts E, Aminian A, Angrisani L, Cohen RV, et al. American society for metabolic and bariatric surgery (ASMBS) and international federation for the surgery of obesity and metabolic disorders (IFSO): indications for metabolic and bariatric surgery. Surg Obes Relat Dis. 2022;18:1345–56. 36280539 10.1016/j.soard.2022.08.013

[3] Mohamedahmed AYY, Hamid M, Zaman S, Abdalla HE, Wuheb AA, Khan A, et al. Does omentopexy make a difference in laparoscopic sleeve gastrectomy for obesity treatment? A systematic review and meta-analysis. Obes Surg. 2023. 10.1007/s11695-023-06956-x 38038906

[4] English WJ, DeMaria EJ, Hutter MM, Kothari SN, Mattar SG, Brethauer SA, et al. American Society for Metabolic and Bariatric Surgery 2018 estimate of metabolic and bariatric procedures performed in the United States. Surg Obes Relat Dis [Internet]. 2020;16:457–63. Available from: https://www.sciencedirect.com/science/article/pii/S1550728919311608 32029370 10.1016/j.soard.2019.12.022

[5] Wozniewska P, Diemieszczyk I, Hady HR. Complications associated with laparoscopic sleeve gastrectomy - A review. Prz Gastroenterol. 2021;5–9. 10.5114/pg.2021.104733 PMC8112272 33986881

[6] Banescu B, Balescu I, Copaescu C. Postoperative bleeding risk after sleeve gastrectomy. A two techniques of stapled line reinforcement comparative study in 4996 patients. Chirurgia (Romania). 2019;114:693–703. 10.21614/chirurgia.114.6.693 31928574

[7] Mocanu V, Dang J, Ladak F, Switzer N, Birch DW, Karmali S. Predictors and outcomes of bleed after sleeve gastrectomy: an analysis of the MBSAQIP data registry. Surg Obes Relat Dis. 2019;15:1675–81. 31590999 10.1016/j.soard.2019.07.017

[8] Zafar SN, Felton J, Miller K, Wise ES, Kligman M. Staple line treatment and bleeding after laparoscopic sleeve gastrectomy. J Soc Laparoendosc Surg. 2018;22. 10.4293/JSLS.2018.00056 PMC6305063 30607100

[9] Golzarand M, Toolabi K, Parsaei R. Prediction factors of early postoperative bleeding after bariatric surgery. Obes Surg. 2022;32:1–8. 35474043 10.1007/s11695-022-06059-z

[10] Gagner M, Buchwald JN. Comparison of laparoscopic sleeve gastrectomy leak rates in four staple-line reinforcement options: A systematic review. Surg Obes Relat Dis. 2014;10:713–23. 24745978 10.1016/j.soard.2014.01.016

[11] Caiazzo R, Marciniak C, Wallach N, Devienne M, Baud G, Cazauran JB, et al. Malignant leakage after sleeve gastrectomy: endoscopic and surgical approach. Obes Surg. 2020;30:4459–66. 32623688 10.1007/s11695-020-04818-4

[12] Zarzycki P, Kulawik J, Małczak P, Rubinkiewicz M, Wierdak M, Major P. Laparoscopic sleeve gastrectomy with omentopexy: is it really a promising method?-a systematic review with meta-analysis. Available from: 10.1007/s11695-021-05327-8 10.1007/s11695-021-05327-8 PMC8113139 33677783

[13] Rosenthal RJ. International sleeve gastrectomy expert panel consensus statement: Best practice guidelines based on experience of >12,000 cases. Surg Obes Relat Dis. 2012;8:8–19. 22248433 10.1016/j.soard.2011.10.019

[14] Gagner M, Deitel M, Erickson AL, Crosby RD. Survey on laparoscopic sleeve gastrectomy (LSG) at the fourth international consensus summit on sleeve gastrectomy. Obes Surg. 2013;23:2013–7. 23912263 10.1007/s11695-013-1040-x

[15] Aiolfi A, Gagner M, Zappa MA, Lastraioli C, Lombardo F, Panizzo V, et al. Staple line reinforcement during laparoscopic sleeve gastrectomy: systematic review and network meta-analysis of randomized controlled trials. Obes Surg. 2022;32:1466–78. 35169954 10.1007/s11695-022-05950-z PMC8986671

[16] Kara YBIIESYSYS. ultrasonography guided modified BRILMA (Blocking the cutaneous branches of intercostal nerves in the middle axillary line) block in bariatric surgery. J Laparoendosc Adv Surg Tech Videoscop. 2023. 10.1089/lap.2023.0223 37787937

[17] Sabry K, Qassem M. The impact of routine omentopexy to staple line on the incidence of early postoperative complications after laparoscopic sleeve gastrectomy: is it worth? Egypt J Surg. 2018;37:479. 10.4103/ejs.ejs_56_18

[18] Abou-Ashour HS. Impact of gastropexy/omentopexy on gastrointestinal symptoms after laparoscopic sleeve gastrectomy. Obes Surg. 2022;32:729–36. 34870791 10.1007/s11695-021-05806-y PMC8866353

[19] Abosayed AK, Mostafa MS. Omentopexy effect on the upper gastrointestinal symptoms and the esophagogastroduodenoscopy findings in patients undergoing sleeve gastrectomy. Obes Surg. 2022;32:1864–71. 35320488 10.1007/s11695-022-05995-0 PMC9072512

[20] Nosrati SS, Pazouki A, Sabzikarian M, Pakaneh M, Kabir A, Kermansaravi M. Can omentopexy reduce the incidence of gastroesophageal reflux disease after laparoscopic sleeve gastrectomy. Obes Surg. 2021;31:274–81. 32809139 10.1007/s11695-020-04923-4

[21] Arslan E, Banli O, Sipahi M, Yagci G. Effects and results of omentopexy during laparoscopic sleeve gastrectomy [Internet]. 2018. Available from: www.surgical-laparoscopy.com 10.1097/SLE.0000000000000526 29668667

[22] Diab ARF, Sher T, Awshah S, Noom M, Docimo S, Sujka JA, et al. Oversewing/suturing of the staple line during sleeve gastrectomy is an effective and affordable staple line reinforcement method: a meta-analysis of randomized controlled trials. Obes Surg. 2023:2533–45. 10.1007/s11695-023-06672-6 37312007

[23] Iossa A, Abdelgawad M, Watkins BM, Silecchia G. Leaks after laparoscopic sleeve gastrectomy: overview of pathogenesis and risk factors. Langenbecks Arch Surg. 2016:757–66. 10.1007/s00423-016-1464-6 27301373

[24] Jurowich C, Thalheimer A, Seyfried F, Fein M, Bender G, Germer CT, et al. Gastric leakage after sleeve gastrectomy-clinical presentation and therapeutic options. Langenbecks Arch Surg. 2011;396:981–7. 21556930 10.1007/s00423-011-0800-0

[25] Khoursheed M, Al-Bader I, Mouzannar A, Ashraf A, Bahzad Y, Al-Haddad A, et al. Postoperative bleeding and leakage after sleeve gastrectomy: a single-center experience. Obes Surg. 2016;26:2944–51. 27277092 10.1007/s11695-016-2215-z

[26] Al-Rashedy M, Ghosh A, Mukherjee T, Halai S, Mahmood RA, Krivan S, et al. The role of relaparoscopy in the management of early bariatric surgery complications and 30-day outcome: a tertiary centre experience. Available from: 10.1007/s11695-021-05401-1 10.1007/s11695-021-05401-1 33881739

[27] Wood SG, Kumar SB, Dewey E, Lin MY, Carter JT. Safety of concomitant cholecystectomy with laparoscopic sleeve gastrectomy and gastric bypass: a MBSAQIP analysis. Surg Obes Relat Dis. 2019;15:864–70. 31060907 10.1016/j.soard.2019.03.004

[28] de Lucena AVS, Cordeiro GG, Leão LHA, Kreimer F, de Siqueira LT, da Conti Oliveira Sousa G, et al. Cholecystectomy concomitant with bariatric surgery: safety and metabolic effects. Obes Surg. 2022;32:1093–102. 35064462 10.1007/s11695-022-05889-1

[29] Yardimci S, Coskun M, Demircioglu S, Erdim A, Cingi A. Is concomitant cholecystectomy necessary for asymptomatic cholelithiasis during laparoscopic sleeve gastrectomy? Obes Surg. 2018;28:469–73. 28803397 10.1007/s11695-017-2867-3

[30] Clapp B, Liggett E, Barrientes A, Aguirre K, Marwaha V, Tyroch A. Concomitant hiatal hernia repair with sleeve gastrectomy: A 5-year analysis. J Soc Laparoendosc Surg. 2020;24. 10.4293/JSLS.2020.00066 PMC7732366 33414611

[31] Clapp B. Comment on: Sleeve gastrectomy with concomitant hiatal hernia repair in obese patients: long-term results on gastroesophageal reflux disease. In: Surgery for obesity and related diseases: Elsevier Inc; 2020. p. 1177–8. 10.1016/j.soard.2020.05.027 32653366

